# The role of the mitochondrial injury-mediated oxidative stress-sterile inflammation axis in varicocele-induced infertility: a narrative review

**DOI:** 10.1186/s12610-026-00324-2

**Published:** 2026-10-22

**Authors:** Jun Zhang, Boxian Gao, Yue Zhang, Jun Guo, Chongfu Zhong

**Affiliations:** 1 The First Clinical College, Shandong University of Traditional Chinese Medicine, Jinan, 250014 China; 2 Department of Peripheral Vascular Diseases, Affiliated Hospital of Shandong University of Traditional Chinese Medicine, Jinan, 250014 China; 3 Department of Andrology, Xiyuan Hospital of China Academy of Chinese Medical Sciences, Beijing, 100091 China; 4 Department of Andrology, Affiliated Hospital of Shandong University of Traditional Chinese Medicine, Jinan, 250014 China

**Keywords:** Varicocele, Male infertility, Mitochondrial dysfunction, Mitochondrial DNA, CGAS-STING signaling, NLRP3 inflammasome, Varicocèle, Infertilité masculine, Dysfonction mitochondriale, ADN mitochondrial, Signalisation cGAS-STING, Inflammasome NLRP3

## Abstract

Varicocele (VC) is a common correctable factor in male infertility, yet its effect on fertility and response to repair vary markedly among patients. This narrative review examines whether mitochondrial injury links the hemodynamic lesion to oxidative stress, sterile inflammation, and persistent spermatogenic damage. Venous stasis, hyperthermia, hypoxia, and metabolic disturbance can impair mitochondrial respiration, membrane potential, ATP production, and quality control. These changes increase mitochondrial reactive oxygen species and favor mitochondrial DNA (mtDNA) oxidation and release. Oxidized mtDNA can engage cyclic GMP-AMP synthase (cGAS)-stimulator of interferon genes (STING) and NOD-like receptor family pyrin domain-containing 3 (NLRP3) signaling in nucleated testicular cells, but the evidence is uneven. Human studies mainly document sperm mitochondrial dysfunction, oxidative injury, DNA fragmentation, and altered inflammasome-related factors in seminal plasma. The integrated mtDNA-cGAS-STING-NLRP3 sequence is supported chiefly by animal and non-VC mechanistic models and has not been causally demonstrated in human VC. Anatomical and temporal context is therefore critical. Events in testicular somatic, germ, and immune cells during spermatogenesis cannot be inferred from epididymal, ejaculated-sperm, seminal-plasma, or female-tract findings. Endoplasmic reticulum stress and autophagy or mitophagy may modify mitochondrial injury, whereas programmed cell death, blood-testis barrier disruption, and epigenetic alterations may extend its consequences. Most of these links still require cell-resolved and longitudinal validation. Guideline-directed varicocele repair remains the clinical foundation. Sperm DNA fragmentation and other mechanism-related markers are useful research tools but lack source specificity or validated treatment thresholds, and pathway-directed therapies remain preclinical. The distinctive contribution of this review is a compartment-resolved and evidence-graded model that separates demonstrated findings from plausible links. It also defines the experiments needed to determine whether residual mitochondrial-inflammatory injury predicts reproductive outcomes or response to safe adjunctive treatment.

## Résumé

Le varicocèle (VC) est un facteur fréquent et curable d’infertilité masculine, mais son impact sur la fertilité et la réponse à la correction varient considérablement d’un patient à l’autre. Cette revue narrative examine dans quelle mesure une atteinte mitochondriale pourrait relier la lésion hémodynamique au stress oxydatif, à l’inflammation stérile et à des altérations spermatogénétiques persistantes. La stase veineuse, l’hyperthermie, l’hypoxie et les désordres métaboliques peuvent altérer la respiration mitochondriale, le potentiel de membrane, la production d’ATP et les mécanismes de contrôle de qualité. Ces perturbations augmentent la production d’espèces réactives de l’oxygène d’origine mitochondriale et favorisent l’oxydation et la libération de l’ADN mitochondrial (ADNmt). L’ADNmt oxydé peut activer les voies de signalisation de la cyclic GMP-AMP synthase (cGAS)–stimulator of interferon genes (STING) et du NOD-like receptor family pyrin domain-containing 3 (NLRP3) dans les cellules testiculaires nucléées, mais les données restent hétérogènes. Les études humaines documentent surtout une dysfonction mitochondriale des spermatozoïdes, des lésions oxydatives, une fragmentation de l’ADN et des modifications de facteurs liés à l’inflammasome dans le plasma séminal. La séquence intégrée ADNmt–cGAS–STING–NLRP3 est principalement étayée par des modèles animaux et des modèles mécanistiques non liés au VC, et n’a pas été démontrée de manière causale dans le varicocèle humain. Le contexte anatomique et temporel est donc déterminant. Les événements survenant dans les cellules somatiques, germinales et immunitaires testiculaires au cours de la spermatogenèse ne peuvent être déduits à partir d’observations portant sur les spermatozoïdes épididymaires ou éjaculés, le plasma séminal ou le tractus génital féminin. Le stress du réticulum endoplasmique et l’autophagie ou la mitophagie peuvent moduler l’atteinte mitochondriale, tandis que la mort cellulaire programmée, la rupture de la barrière hémato-testiculaire et les modifications épigénétiques peuvent en amplifier les conséquences. La plupart de ces liens nécessitent encore une validation à résolution cellulaire et dans une perspective longitudinale. La prise en charge du varicocèle fondée sur les recommandations reste le socle clinique. La fragmentation de l’ADN spermatique et d’autres biomarqueurs liés aux mécanismes étudiés constituent des outils de recherche utiles, mais ils manquent de spécificité quant à leur origine et ne disposent pas de seuils thérapeutiques validés; les traitements ciblant spécifiquement ces voies demeurent au stade préclinique. L’apport original de cette revue est de proposer un modèle compartimentalisé et gradué en fonction du niveau de preuve, qui distingue clairement les résultats établis des liens plausibles. Elle définit également les expériences nécessaires pour déterminer si des lésions mitochondriales et inflammatoires résiduelles permettent de prédire le pronostic reproductif ou la réponse à des traitements adjuvants sûrs.

## Introduction

Varicocele (VC) is characterized by dilatation and tortuosity of the pampiniform plexus and by venous reflux. It is among the most common correctable factors in male infertility. Current guidelines recommend considering repair in men attempting conception when a palpable clinical VC coexists with infertility and abnormal semen parameters [1, 2]. The diagnosis alone, however, does not predict the degree of reproductive impairment. Some men with clinical VC remain fertile, and improvement after repair is variable. Contemporary guidelines therefore base treatment on the clinical and reproductive context rather than venous anatomy alone [1, 2]. Consistent with this heterogeneity, Pasqualotto et al. reported different semen-quality and oxidative-stress profiles in fertile and infertile men with VC, although baseline testicular reserve was not measured [3]. Reviews of VC pathophysiology consistently describe a multifactorial process and conclude that no single mechanism explains its variable reproductive effect [4–7]. Individual reproductive potential is therefore likely to modify the clinical expression of a similar venous lesion [3–7].

Against this clinical background, mechanistic research has identified several interacting stressors rather than a single downstream pathway. Scrotal hyperthermia, impaired testicular perfusion and hypoxia, reflux of adrenal or renal metabolites, and endocrine dysregulation can disturb redox and immune homeostasis, weaken the blood-testis barrier (BTB), and damage sperm DNA [4–7]. Reactive oxygen species (ROS) are required for sperm capacitation and the acrosome reaction. In excess, however, they promote membrane lipid peroxidation, protein oxidation, mitochondrial dysfunction, and DNA strand breaks [8–11]. Human and animal studies of VC have also reported increased inflammatory mediators, altered components of the NOD-like receptor family pyrin domain-containing 3 (NLRP3) inflammasome, and impaired testicular immune homeostasis [12–15]. These observations shift the central question from whether oxidative stress and inflammation contribute to VCI to how mitochondrial injury might connect and sustain them.

This review places mitochondria at the center of this network and proposes a mitochondrial injury-mediated oxidative stress-sterile inflammation axis. Rather than treating oxidative stress, mitochondrial dysfunction, inflammation, BTB injury, and epigenetic change as parallel observations, we ask whether mitochondrial injury provides a mechanistic bridge among them [4–8, 12]. We trace each link across anatomical compartments and stages of sperm development. We also separate evidence from human VC, experimental VC, and mechanistically related non-VC models, and identify which claims remain untested. By combining a compartment-resolved map with an explicit evidence hierarchy, this review turns a broad biological hypothesis into testable questions for biomarker, mechanistic, and intervention studies.

## Literature search

This narrative review was updated through 25 August 2026. We searched PubMed/MEDLINE, Web of Science Core Collection, Scopus, and Embase from database inception. Search terms combined ‘varicocele’ or ‘varicocoele’ with male infertility, testis, sperm, mitochondria, mitochondrial DNA, oxidative stress, endoplasmic reticulum stress, unfolded protein response, autophagy, mitophagy, cGAS-STING, NLRP3, inflammasome, pyroptosis, blood-testis barrier, DNA methylation, histone, protamine, chromatin, noncoding RNA, m6A, proteomics, metabolomics, single-cell, and spatial transcriptomics. Eligible sources included peer-reviewed human studies, experimental VC studies, and reproductive-cell studies with direct mechanistic relevance. Clinical guidelines and systematic or high-quality narrative reviews were used for contextual and synthetic claims. We prioritized original VC studies and retained supportive, discordant, and negative findings. Conference abstracts without adequate methods and nonreproductive studies without a direct mechanistic link were excluded. Reference lists of eligible papers were also checked. The final synthesis included 93 references. Screening was not duplicated, risk of bias was not formally assessed, and no protocol was registered. This work should therefore be interpreted as a narrative review rather than a systematic review or meta-analysis.

## Mitochondrial injury and cellular stress responses in VCI

### Hemodynamic stress and sperm mitochondrial dysfunction

Impaired spermatic venous return is the initiating alteration in VC. Persistent venous hypertension, blood stasis, local hyperthermia, and hypoxia can disturb the testicular microenvironment and increase oxidative burden [16]. Mitochondria are concentrated in the sperm midpiece. Although the relative contributions of oxidative phosphorylation and glycolysis vary across species and stages of sperm motility, mitochondria directly support adenosine triphosphate (ATP) production, redox homeostasis, calcium signaling, and apoptotic regulation. Electron transport chain impairment increases electron leakage and mtROS generation. This creates a positive-feedback loop of declining mitochondrial function, ROS accumulation, and further mitochondrial injury. Clinical studies provide associative evidence for this process. Sperm mitochondrial respiratory activity is lower in men with VC than in healthy controls and is accompanied by reduced sperm concentration and motility [17]. Other studies have reported abnormal chromatin packaging, reduced mitochondrial membrane potential (ΔΨm), and a higher proportion of apoptotic spermatozoa [18]. A cross-sectional study also found lower ΔΨm and ATP levels, together with increased apoptosis and DNA fragmentation index (DFI), in men with VCI. These measures correlated with conventional semen parameters [19]. Potential confounders were not adequately controlled, however. The findings therefore establish association rather than the causal direction between VCI and mitochondrial dysfunction. Proteomic studies have likewise identified abnormalities in proteins involved in the electron transport chain, energy metabolism, membrane integrity, and redox regulation in spermatozoa and sperm mitochondrial fractions from men with VCI [20, 21]. Collectively, these findings support mitochondrial dysfunction as an important component of VCI. Its temporal sequence and independent contribution to clinical outcomes require longitudinal validation. Blumer et al. found reduced sperm mitochondrial activity and increased sperm DNA fragmentation in men with VC, whereas lipid peroxidation did not differ significantly from controls [22]. In an experimental rat time-course, Minas et al. observed impaired sperm mitochondrial activity at both 2 and 4 months, without further worsening at the longer interval [23]. These original studies reinforce the mitochondrial phenotype while also illustrating small samples and temporal or individual heterogeneity.

### Anatomical compartments and timing of injury

The biological meaning of mitochondrial injury depends on where and when it occurs. During spermatogenesis, developing germ cells coexist with nucleated Sertoli, Leydig, endothelial, and immune cells in distinct microenvironments. Non-VC models show that heat stress can activate NLRP3 in Sertoli cells and that impaired mitophagy with mtDNA release can engage cGAS-STING signaling in Leydig cells [24, 25]. These studies establish cellular plausibility, not the same sequence in VC. During epididymal transit, sperm encounter a separate epithelial and luminal environment [26]. Experimental VC causes ATF6-dependent epididymal epithelial inflammation, but local NLRP3 assembly has not been demonstrated [27]. After ejaculation, seminal plasma contains extracellular vesicles from several male reproductive tissues, which prevents testicular or sperm source attribution without fractionation [28]. In the female tract, mitochondrial and ROS signaling contributes to normal capacitation [29], but no VC-specific DAMP-inflammasome pathway has been shown. We therefore apply the central mtDNA-sensing model mainly to injured nucleated cells within the testis. Ejaculated sperm and seminal markers are treated as downstream readouts unless source-resolved evidence is available.

### mtDNA release and metabolic signaling

The consequences of mitochondrial injury extend beyond inadequate energy production. When mitochondrial membrane permeability increases, membrane integrity is lost, or mitochondrial quality control fails in injured somatic or immune cells, mtDNA can enter the cytosol. It can subsequently be released into the extracellular space through cell death, vesicular transport, or other routes [30, 31]. The absence of histone protection and the proximity of mtDNA to sites of ROS production make it particularly susceptible to oxidative modification. Oxidized mtDNA can therefore serve as both a marker of injury and an immunostimulatory molecule. The sequence of increased mtROS, mtDNA oxidation, and mtDNA release represents a candidate intermediate step that converts oxidative stress into sterile inflammation. Mitochondria-associated metabolites, including ATP, nicotinamide adenine dinucleotide (NAD+), acetyl coenzyme A (acetyl-CoA), α-ketoglutarate (α-KG), and S-adenosylmethionine (SAM), can also influence enzymes that modify DNA, histones, and RNA [32, 33]. This provides a plausible biological link between mitochondrial dysfunction and epigenetic alterations, although direct evidence in VCI remains insufficient.

Human studies provide preliminary evidence linking mitochondrial injury with epigenetic alterations. Arya et al. compared 40 men with clinical VCI and 30 fertile controls. Men with VCI had lower sperm ΔΨm, higher intracellular ROS levels and mtDNA copy numbers, and abnormal promoter methylation in several genes related to mitochondrial structure or function. Three months after varicocele repair or antioxidant treatment, ROS levels and mtDNA copy numbers decreased. ΔΨm did not recover, and methylation changes at several CpG sites persisted [34]. The study had a limited sample size, included heterogeneous interventions, and had a short follow-up period. Pregnancy and live birth were not assessed. It therefore suggests, but does not establish, that persistent mitochondrial and epigenetic abnormalities may contribute to incomplete recovery after treatment.

### ER stress, autophagy, and mitochondrial quality control

Endoplasmic reticulum (ER) stress forms a second stress-response network that can converge on mitochondria. Misfolded proteins activate the unfolded protein response through IRE1, PERK, and ATF6. Early signaling supports protein folding and redox control, whereas persistent IRE1-JNK or PERK-ATF4-CHOP activity can disturb calcium homeostasis, increase NF-κB signaling and ROS production, and promote mitochondrial apoptosis. A rat VC study demonstrated IRE1/XBP1s/JNK activation with increased caspase-3, and a broader review supports bidirectional ER-mitochondrial crosstalk in male reproductive injury [35, 36]. Human VC evidence remains insufficient. ER stress is therefore an experimentally supported modifier, not a validated human component of the proposed axis.

These ER-mitochondrial interactions are shaped by autophagy, particularly the selective clearance of damaged mitochondria. Effective mitophagy limits mtROS accumulation, mtDNA escape, inflammasome activation, and apoptosis. Incomplete or stalled flux allows these signals to persist. In a rat VC model, ATG7 and the LC3-II/LC3-I ratio increased in testis and epididymal sperm together with oxidative injury. A small human case-control study likewise found higher sperm ATG7 and LC3 alongside apoptosis and protamine deficiency [37, 38]. Both studies relied mainly on static markers. They show recruitment of autophagy-related machinery but do not establish whether flux was protective, insufficient, or blocked. Time-resolved flux assays and pathway-specific rescue are therefore needed before mitophagy failure can be placed upstream of mtDNA release in VCI.

## Immune sensing of oxidized mtDNA: cross-amplification between cGAS-STING and NLRP3

mtDNA is relatively enriched in unmethylated CpG motifs and therefore has immunostimulatory properties similar to those of bacterial DNA. After mitochondrial injury, oxidized mtDNA in the cytosol or extracellular space can be recognized as a DAMP by the innate immune system. This recognition converts intracellular oxidative damage into inflammatory signaling.

Cytosolic mtDNA can be recognized by cyclic GMP-AMP synthase (cGAS), which generates cyclic GMP-AMP (cGAMP). cGAMP activates stimulator of interferon genes (STING), TANK-binding kinase 1 (TBK1), interferon regulatory factor 3 (IRF3), and nuclear factor-κB (NF-κB), leading to type I interferon and proinflammatory signaling [31, 39]. NLRP3 activity is best understood as a staged process. NF-κB-dependent priming increases NLRP3 and pro-interleukin-1β (pro-IL-1β) expression and establishes post-translational competence. A second signal, including potassium efflux and NEK7 engagement, extracellular ATP-P2×7 signaling, calcium disturbance, lysosomal injury, mtROS, or oxidized mtDNA, can then promote inflammasome assembly [40, 41]. The assembled NLRP3-apoptosis-associated speck-like protein containing a CARD (ASC)-caspase-1 complex produces mature interleukin-1β (IL-1β) and interleukin-18 (IL-18) and cleaves gasdermin D (GSDMD) to induce pyroptosis [42, 43]. This sequence also defines the evidence needed to claim activation. Increased total NLRP3 or caspase-1 indicates pathway availability but not complex assembly. ASC specks, cleaved caspase-1 and GSDMD, mature cytokines, and pathway-specific rescue provide stronger evidence [40, 41]. cGAS-STING and NLRP3 are not a simple linear cascade. They can reinforce one another through NF-κB-dependent priming, mitochondrial injury, and cytokine signaling [44–46]. In VCI, they are therefore considered a potential cross-amplifying network rather than an established unidirectional pathway.

The strength of VC-related evidence differs substantially across this proposed network. In a rat model of VC, Jia et al. found that chlorogenic acid improved mitochondrial homeostasis, reduced the release of oxidized mtDNA, and suppressed cGAS-STING- and NLRP3-related signaling. These changes were accompanied by less severe spermatogenic impairment [47]. This study provides direct animal evidence in VC for a framework linking mitochondrial injury, oxidized mtDNA, DNA sensing, and inflammasome activation. Nevertheless, the pleiotropic effects of a pharmacological intervention cannot substitute for genetic validation of causality. In a non-VC model of testicular injury, deoxynivalenol induced mitochondrial injury and cytosolic mtDNA leakage, followed by activation of cGAS-STING-TBK1/IRF3 and NF-κB signaling [39]. These findings provide mechanistic support for sterile inflammation within the testis but are not direct evidence in VC. Human data focus mainly on inflammasome components. Baazm et al. reported increased NLRP3, ASC, and IL-1β in the seminal plasma of 32 men with grade 2 or 3 VC. IL-18 was lower, however, and caspase-1 did not differ significantly [48]. Thus, changes in seminal inflammasome-related components were not uniform. Possible explanations include measurement of total rather than cleaved caspase-1, differential processing or release of IL-18 and IL-1β, accessory-gland or leukocyte contributions to seminal plasma, limited statistical power, and pathway activity in cell populations not resolved by bulk semen. The study therefore supports an altered seminal inflammatory milieu but does not demonstrate coordinated canonical NLRP3 activation [41]. Camargo et al. also reported altered seminal inflammasome activity in adults with VC [49]. In animal models of VC, NLRP3, caspase-1, and IL-1β are upregulated, whereas inhibition of related signaling reduces testicular injury and improves sperm quality [50–52]. Overall, evidence for NLRP3 involvement in VC-related inflammation is comparatively strong. Direct links among mtDNA, cGAS-STING, and NLRP3 in human VCI remain unproven.

These findings support a testable working model. Venous stasis, heat stress, and hypoxia first disturb mitochondrial homeostasis and promote mtROS accumulation. mtDNA is then oxidized and released abnormally, activating DNA-sensing and inflammasome pathways. The release of inflammatory mediators and inflammatory cell death can, in turn, aggravate mitochondrial injury. This model emphasizes bidirectional amplification between oxidative stress and sterile inflammation. It should not be interpreted as a fixed pathway already validated in human populations.

## Downstream consequences of the mitochondrial-inflammatory axis: programmed cell death and microenvironmental disruption

This inflammatory network can extend mitochondrial injury into persistent impairment of spermatogenesis through multiple forms of programmed cell death, compromised BTB function, and remodeling of the testicular immune microenvironment.

### Programmed cell death

Apoptosis is the best-characterized form of cell death in VC-related spermatogenic injury. Ferroptosis, pyroptosis, and necroptosis have recently attracted attention as additional mechanisms. These processes can be initiated by oxidative stress, mitochondrial injury, or inflammatory signaling. They can also induce secondary inflammation through the release of DAMPs. Their cellular origins, temporal sequence, and relative contributions in VCI have not been established.

In mitochondrial apoptosis, reduced ΔΨm, an increased BCL2-associated X protein (BAX)/B-cell lymphoma 2 (BCL-2) ratio, cytochrome c release, and activation of the caspase cascade can promote apoptosis in germ cells or spermatozoa [18, 19]. Mature spermatozoa contain little cytoplasm and have limited DNA repair capacity. They are therefore particularly vulnerable to mitochondrial injury and ROS accumulation. VC animal models and in vitro oxidative-stress studies have shown increased ROS, endoplasmic reticulum stress, apoptosis, and sperm abnormalities [53]. In mice with VC, lycopene reduced spermatocyte apoptosis through a miR-23a/b-PROK2-related mechanism [54]. These intervention studies support a role for apoptosis in VC-related injury but do not establish that it is the dominant mechanism in every patient.

Ferroptosis is driven by iron-dependent peroxidation of membrane lipids. Typical features include increases in labile iron and lipid ROS, upregulation of acyl-CoA synthetase long-chain family member 4 (ACSL4), and failure of glutathione peroxidase 4 (GPX4)-mediated defense [55]. Sperm membranes are rich in polyunsaturated fatty acids and are therefore susceptible to ferroptosis-like lipid peroxidation. Whether mature spermatozoa execute the canonical ferroptotic program of nucleated cells remains debated. In the absence of morphological evidence, pharmacological rescue, and confirmation with multiple markers, the term “ferroptosis-related changes” is more appropriate than a definitive diagnosis of ferroptosis.

Pyroptosis is an inflammatory form of cell death mediated by pore formation by gasdermin proteins. The canonical NLRP3-caspase-1 pathway primarily cleaves GSDMD and promotes the maturation and release of IL-1β and IL-18. GSDME is usually cleaved by caspase-3 and can convert some apoptotic signals into pyroptosis-like membrane rupture [40, 46]. Activation of the NLRP3/caspase-1/GSDMD pathway has been observed in rats with VC and in seminiferous tubule-associated cells cultured under hypoxia. Blocking this pathway reduces testicular injury, improves spermatogenesis, and protects Sertoli cells [51]. Because both drug-containing serum and multicomponent formulations can act on multiple targets, cell-specific knockout or selective inhibition is still required to establish causality.

A human sperm study found increased ROS and iron levels in infertile men with bilateral VC. Glutathione, total superoxide dismutase, and catalase levels were reduced, and GPX4, receptor-interacting serine/threonine-protein kinase 1/3 (RIPK1/3), and GSDME were altered [56]. These findings suggest that ferroptosis-related changes, necroptosis, and GSDME-related inflammatory death can coexist. Cross-sectional differences in protein expression, however, are insufficient to demonstrate the complete execution of multiple cell-death programs. Ferroptosis- and N6-methyladenosine (m6A)-related changes have also been observed in cremaster muscle satellite cells from rats with VC [57]. Because the tissue of origin differs, these findings cannot be directly extrapolated to the testis or spermatozoa.

### BTB disruption and remodeling of the testicular immune microenvironment

The BTB is formed by tight junctions, basal ectoplasmic specializations, gap junctions, and other structures between adjacent Sertoli cells. It is essential for maintaining the spermatogenic microenvironment and restricting the entry of immune components into the seminiferous tubules [58, 59]. The BTB is not a static barrier. It undergoes continuous remodeling during the seminiferous epithelial cycle. VC-related heat stress, oxidative stress, and inflammation can shift this dynamic balance toward pathological hyperpermeability.

ROS and proinflammatory mediators can weaken Sertoli cell junctions and BTB integrity by altering junctional proteins, the actin cytoskeleton, and related kinase signaling [16, 58, 60, 61]. Mitochondrial function, antioxidant capacity, lactate supply, and phagocytic clearance by Sertoli cells also directly support germ cell survival. Sertoli cell injury can therefore compromise both barrier function and nutritional support. Leydig cells are responsible for testosterone synthesis. In a non-VC mouse model induced by deoxynivalenol, ROS-NLRP3-caspase-1-GSDMD signaling promoted Leydig cell pyroptosis and disrupted testicular endocrine homeostasis [62]. This finding demonstrates that Leydig cells are capable of such a response, but it does not establish the same process in VC.

VC is a noninfectious hemodynamic disorder, and its inflammatory response is therefore more consistent with sterile inflammation. Endogenous danger signals, including oxidized mtDNA, high-mobility group box 1, heat-shock proteins, and lipid peroxidation products, can activate pattern-recognition receptors in Sertoli cells, germ cells, and testicular immune cells [47, 58]. Increased BTB permeability can also expose germ cell antigens and alter the state of local immune cells. Mitochondrial injury and barrier disruption can thus form a mutually reinforcing loop. The cell type that initiates this loop remains unknown.

## Epigenetic alterations: a potential long-term molecular imprint rather than an established outcome

Epigenetic alterations can provide a long-term molecular record linking metabolic state, environmental stress, and gene expression. Changes in NAD+, acetyl-CoA, α-KG, and SAM caused by mitochondrial dysfunction can influence DNA methyltransferases, histone acetyltransferases or deacetylases, and RNA-modifying enzymes [32, 33]. Oxidative stress and inflammation can also damage DNA, disrupt repair, and alter sperm chromatin packaging. Most evidence for these metabolic-epigenetic relationships comes from other diseases or basic models. They cannot yet be regarded as an established causal chain in VCI.

A recent synthesis organizes VC-associated epigenetic evidence into DNA methylation, chromatin packaging, noncoding RNA, and m6A-RNA regulation, while emphasizing the limited longitudinal and offspring-level evidence [63]. Case-control studies have identified differential sperm DNA methylation and increased expression of the de novo DNA methyltransferases DNMT3A and DNMT3B in men with VC [64, 65]. During spermatogenesis, histones are progressively replaced by protamines to compact the paternal genome. Histone retention is increased in men with VC and has been reported to decline after microsurgical varicocelectomy, while increased protamine defects have been observed in rats with VC [66, 67]. These measures indicate incomplete chromatin packaging. They should not be interpreted as locus-specific histone modifications. Direct VC evidence for histone acetylation or methylation at defined loci, chromatin accessibility, and three-dimensional chromatin organization remains sparse. Noncoding-RNA studies have reported increased expression of the hypoxia-related long noncoding RNAs (lncRNAs) MIR210HG and MLLT4-AS1, with correlations to ROS and semen parameters [68]. Seminal-plasma MIR210HG is also associated with VC severity and spermatogenic impairment and has been reported to decrease six months after repair [69]. lncRNA gadd7, SLC7A11-AS1 isoforms, and several microRNAs have been linked to apoptosis, oxidative stress, or immune regulation [70–72]. In rats with VC, testicular expression of m6A-related enzymes is altered, and m6A-dependent regulation of PLOD2 has been implicated in spermatocyte injury [73, 74]. Enzyme abundance alone, however, neither identifies the modified transcripts nor establishes functional causality [63, 73, 74]. Most studies are small or experimental, and cross-cohort replication is limited. Future work should combine purified sperm or cell-resolved testicular samples with bisulfite sequencing, retained-histone mapping, chromatin-accessibility assays, and transcript-specific m6A validation.

It is biologically plausible to interpret persistent epigenetic abnormalities after VC repair as residual molecular injury, but this remains a hypothesis. Future studies should use longitudinal preoperative and postoperative designs. They should distinguish the effects of surgery, antioxidant therapy, and lifestyle and relate sperm epigenetic changes to embryo development, pregnancy, and live birth (Table 1).

**Table 1:** Evidence-source summary for the proposed mitochondrial injury-sterile inflammation network

Proposed link	Human evidence	VC animal/in vitro evidence	Critical interpretation
Mitochondrial dysfunction and oxidative injury	Several small cross-sectional sperm studies; limited longitudinal repair data [17–22]	Consistent redox, bioenergetic, and histological changes in VC models [23, 47, 53, 67]	Best-supported component, but anatomical source, temporal order, and patient-level causality remain uncertain.
ER stress and autophagy/mitophagy	One small human sperm study of static autophagy markers; no cell-resolved human ER-stress validation [38]	Rat studies show testicular UPR activation, ATF6-dependent epididymal inflammation, and increased autophagy markers [27, 35, 37, 53]	Animal-supported association; epididymal inflammation is reported, but local inflammasome assembly, flux, and pathway-specific necessity remain unresolved.
Oxidized mtDNA and cGAS-STING	No direct demonstration in human VC testis; sperm mtDNA-copy findings are associative [34]	One integrated VC rat pharmacology study plus supportive non-VC Sertoli/Leydig models [25, 39, 47]	A plausible intratesticular mechanism, not an established human sperm pathway.
NLRP3 inflammasome	Two small seminal studies with discordant markers; human seminal extracellular vesicles have mixed tissue origins [28, 48, 49]	VC rat studies report NLRP3/caspase-1/GSDMD changes; heat-stressed Sertoli cells show NLRP3 activation [24, 47, 50–52]	Human data support an altered seminal inflammatory milieu, not source-resolved, tissue-localized canonical activation.
Epigenetic alterations	Small sperm studies of DNA methylation, chromatin packaging, and noncoding RNA [34, 64–66, 68–70]	Rat DNA/RNA methylation and transcript studies, including m6A work [67, 71–74]	Associations are vulnerable to confounding; causal and reproductive-outcome relevance is unproven.

Human, animal, and in vitro findings are separated because concordant marker changes do not by themselves establish the same causal pathway across compartments

## Candidate biomarkers and the boundaries of current evidence

Routine semen analysis remains the basis of clinical assessment in VCI, but it does not directly capture sperm DNA integrity, oxidative burden, mitochondrial function, or local inflammatory activity. Mechanism-related biomarkers can complement risk assessment and treatment monitoring. Detectability, however, does not imply clinical actionability. Preanalytical procedures, assay platforms, reference intervals, and incremental predictive value all require standardized validation.

Sperm DNA fragmentation (SDF) is currently among the adjunctive markers closest to clinical translation. Meta-analyses indicate that SDF and seminal malondialdehyde (MDA) decrease after varicocele repair [75, 76]. Improvement in DFI appears most evident at approximately 6 months after surgery [77], and SDF is generally higher in men with VC than in healthy controls [78]. SDF is nevertheless a downstream marker of injury and cannot identify its pathological source on its own. Differences among assays and thresholds also limit interchangeability. ΔΨm, ATP, mitochondrial respiration, mtDNA copy number or cell-free mtDNA, cGAS-STING activation, NLRP3-related components, and markers of programmed cell death are useful in mechanistic research. Most lack validated clinical thresholds, reproducibility data, and evidence for outcome prediction. Existing predictors of treatment response include age, duration of infertility, VC grade, testicular volume, gonadotropin levels, total motile sperm count, and imaging parameters [79]. Proteomics and metabolomics can expand the candidate biomarker space [80, 81]. High-dimensional models must undergo validation in independent external cohorts to avoid overfitting and support clinical implementation (Tables 2 and 3).

**Table 2:** Oxidative, mitochondrial, and DNA-integrity biomarkers

Process	Candidate biomarkers	Sample	Potential application	Evidence and limitations	References
Oxidative injury	ROS, MDA, 8-OHdG, TAC, SOD, GSH, GPx	Semen, seminal plasma, serum	Quantify oxidative burden and help explain lipid peroxidation, elevated SDF, and impaired sperm function.	Supported by numerous clinical studies, but assays, reference intervals, and cutoffs are not standardized; results are sensitive to abstinence, handling, and leukocyte contamination.	[4, 5, 7, 17, 75, 82]
Mitochondrial function	ΔΨm, ATP, oxygen consumption/mitochondrial respiration, mitochondrial proteome	Spermatozoa	Assess sperm energy metabolism and mitochondrial homeostasis; explore postoperative recovery and prognostic value.	Exploratory research markers. Cross-platform comparability is limited, and clinical thresholds are not established.	[17–19, 21, 22, 34]
mtDNA-DAMPs	mtDNA copy number, cell-free mtDNA, oxidized mtDNA	Spermatozoa, seminal plasma, serum	Reflect mitochondrial genome stress or extracellular mtDNA burden and explore association with sterile inflammation.	Evidence is mainly animal/mechanistic. Human cell of origin, normalization, oxidation assays, and contamination control are not standardized.	[30, 31, 34, 47, 83, 84]
DNA integrity	SDF/DFI, TUNEL, SCSA, SCD, Comet assay	Spermatozoa	Assess sperm genetic integrity and support risk stratification and postoperative monitoring.	Closer to clinical translation, but platforms and thresholds vary; interpretation should include semen parameters and partner factors.	[4, 5, 20, 75–78]

**Table 3:** Inflammatory, cell-death, and epigenetic biomarkers

Process	Candidate biomarkers	Sample	Potential application	Evidence and limitations	References
cGAS-STING	cGAS, STING, p-TBK1, p-IRF3, type I interferon-related factors	Testis in animal/cell models; human samples require validation	Test DNA-sensing activation and the link between mtDNA-DAMPs and inflammatory conversion.	Evidence comes mainly from animal models; the complete pathway has not been validated in human VC samples.	[31, 39, 46, 47, 85]
Inflammasome	NLRP3, ASC, cleaved caspase-1, IL-1β, IL-18, GSDMD	Seminal plasma, sperm fractions, testis	Characterize local inflammasome-related phenotypes and associations with semen quality or treatment response.	Few human studies with discordant markers; infection, leukocytes, accessory-gland sources, and cleavage state require control.	[12, 28, 41, 47–50, 86]
Programmed cell death	BAX/BCL-2, caspase-3, GPX4, ACSL4, GSDMD/GSDME, RIPK1/RIPK3/MLKL	Spermatozoa, testis	Assess apoptosis and changes related to ferroptosis, pyroptosis, and necroptosis.	Except for apoptosis, evidence is mainly isolated human associations or animal experiments; convergent morphology, function, and rescue are required.	[51, 55–57, 87]
Epigenetic alterations	DNA methylation, chromatin/protamine measures, m6A enzymes, miRNAs and lncRNAs	Spermatozoa, seminal plasma, testicular tissue	Identify persistent molecular changes and explore prognostic value for reproductive outcomes.	Exploratory evidence; studies are small, replication is limited, and standardized assays plus functional validation are lacking.	[34, 63–65, 68–73]

Abbreviations: 8-OHdG, 8-hydroxy-2’-deoxyguanosine; ACSL4, acyl-CoA synthetase long-chain family member 4; ASC, apoptosis-associated speck-like protein containing a CARD; ATP, adenosine triphosphate; BAX, BCL2-associated X protein; BCL-2, B-cell lymphoma 2; cGAS, cyclic GMP-AMP synthase; Comet, single-cell gel electrophoresis assay; DAMPs, damage-associated molecular patterns; DFI, DNA fragmentation index; GSDMD, gasdermin D; GSDME, gasdermin E; GPx, glutathione peroxidase; GPX4, glutathione peroxidase 4; GSH, glutathione; IL-1β, interleukin-1β; IL-18, interleukin-18; IRF3, interferon regulatory factor 3; lncRNA, long noncoding RNA; m6A, N6-methyladenosine; MDA, malondialdehyde; miRNA, microRNA; MLKL, mixed lineage kinase domain-like protein; mtDNA, mitochondrial DNA; NLRP3, NOD-like receptor family pyrin domain-containing 3; RIPK1/3, receptor-interacting serine/threonine-protein kinase 1/3; ROS, reactive oxygen species; SCD, sperm chromatin dispersion; SCSA, sperm chromatin structure assay; SDF, sperm DNA fragmentation; SOD, superoxide dismutase; STING, stimulator of interferon genes; TAC, total antioxidant capacity; TBK1, TANK-binding kinase 1; TUNEL, terminal deoxynucleotidyl transferase dUTP nick-end labeling; ΔΨm, mitochondrial membrane potential

## Therapeutic implications and future directions

### Guideline-directed care and adjunctive strategies

Clinical translation should begin with correction of the hemodynamic lesion. Varicocele repair remains the standard treatment for appropriately selected men with clinical VCI [1, 2, 88]. By correcting venous reflux, surgery reduces heat, hypoxia, and metabolic stress that can injure mitochondria. Postoperative reductions in sperm DNA fragmentation (SDF) and seminal malondialdehyde are consistent with improvement in oxidative injury [75–78]. Better semen or molecular measures, however, do not guarantee natural pregnancy or live birth, and incomplete recovery does not prove persistent activation of one inflammatory pathway. Any adjunctive strategy should therefore be tested after anatomical correction and against clinically meaningful outcomes. No mtDNA-, cGAS-STING-, NLRP3-, ER stress-, or mitophagy-based test or treatment is ready for routine decision-making.

The next question is whether residual injury after repair can identify patients who benefit from an adjunct. SDF is among the markers closest to clinical use, but it is downstream and cannot identify a mitochondrial, inflammatory, or other source. A 2025 systematic review found a relatively consistent decline in SDF around six months after repair, whereas the overall benefit of antioxidants remained uncertain [82]. Randomized evidence indicates that microsurgery can outperform oral antioxidants alone for selected semen parameters, SDF, and natural pregnancy. A small triple-blind trial also reported improved sperm motility and selected oxidative or inflammatory markers with postoperative astaxanthin [89, 90]. These findings justify standardized perioperative trials, not a uniform antioxidant regimen or an assumption that benefit is mediated by the mtDNA-cGAS-STING-NLRP3 axis. Future trials should prospectively define persistent oxidative burden, mitochondrial dysfunction, or elevated SDF and test whether these measures modify the response to adjunctive treatment.

### Preclinical targets and safety boundaries

The model suggests three preclinical intervention points: preserve mitochondrial respiration and quality control; limit oxidized mtDNA generation or abnormal release; and modulate cGAS-STING, NLRP3, or related cell-death signaling only after the relevant cell type and time window are known. In rats with VC, chlorogenic acid improved mitochondrial homeostasis, reduced oxidized mtDNA-related signaling, and alleviated spermatogenic impairment [47]. A systematic review of experimental animals also confirmed consistent testicular oxidative stress and sperm injury in VC models [91]. These findings provide proof of concept, not a clinical recommendation. The immune-privileged testicular environment also creates a safety constraint. Prolonged or nonselective inhibition of cGAS-STING or NLRP3 could impair antimicrobial defense and tissue homeostasis. Preclinical studies must therefore define target selectivity, dose, treatment window, effects on spermatogenesis, and safety for embryos and offspring.

### Longitudinal validation and source-resolved multi-omics

Human validation requires longitudinal cohorts with paired sampling before and after repair and rigorous exclusion of reproductive tract infection, genetic abnormalities, and other major causes of infertility. Standardized assays should assess semen parameters, SDF, oxidative burden, mitochondrial function, cell-free or oxidized mtDNA, and cGAS-STING/NLRP3-related markers over time. Source attribution is essential. Spermatozoa and seminal plasma are accessible, but testicular origin cannot be assumed without fractionation or orthogonal evidence. Spermatic venous blood or testicular tissue should be obtained only when clinically feasible and ethically appropriate. Integrated single-cell RNA sequencing, spatial transcriptomics, proteomics, metabolomics, and epigenomics can help resolve cellular sources, intercellular communication, and the order of mitochondrial injury, inflammation, and barrier disruption [80, 81, 92, 93]. Candidate biomarkers must then demonstrate analytic reproducibility and external validity in independent cohorts.

### From exploratory phenotypes to clinical utility

Exploratory molecular phenotypes should be treated as models, not patient labels. Oxidative burden, mitochondrial function, inflammasome activity, and DNA integrity can be analyzed as continuous variables or used in data-driven clustering. Any resulting pattern should add predictive value beyond VC grade, testicular volume, and conventional semen parameters. Randomized studies must then determine whether the pattern identifies incremental benefit from an adjunct. Until thresholds, stability, and external validity are established, patients should not be assigned fixed “mitochondrial” or “inflammatory” phenotypes.

In parallel, key causal nodes require cell type-specific manipulation, timed interventions, and rescue experiments. Translational endpoints should extend beyond sperm concentration, motility, or a single molecular marker. They should include natural pregnancy, assisted reproductive outcomes, embryo development, miscarriage, and live birth, with adjustment for female-partner age and fertility factors. The proposed axis can inform precision care only when a mechanistic marker reproducibly predicts outcomes and guides a treatment that provides safe, verifiable benefit.

## Conclusions

Varicocele-induced infertility is not a single downstream consequence of venous dilatation. The strongest evidence supports hemodynamic stress, oxidative imbalance, and sperm mitochondrial dysfunction. Animal models further show that oxidized mtDNA, cGAS-STING, and NLRP3 can converge, but the complete chain has not been established in human VC. ER stress and altered autophagy or mitophagy may influence mitochondrial quality control, while programmed cell death, BTB injury, and epigenetic change can prolong damage. Their roles are likely to vary by cell type, anatomical compartment, and stage of sperm development.

**Fig. 1:**
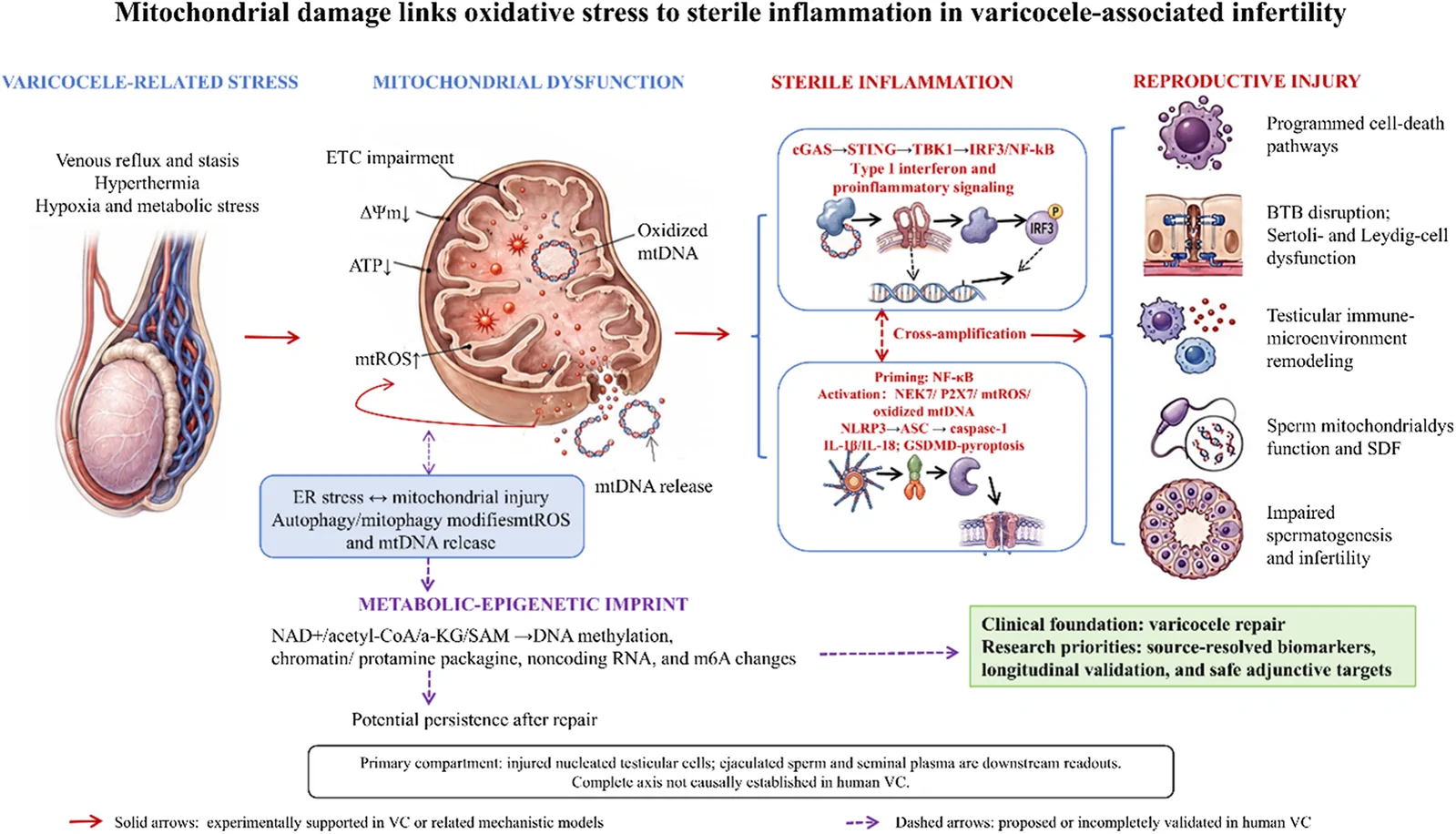
Working model and translational implications of the mitochondrial injury-mediated oxidative stress-sterile inflammation axis in varicocele-induced infertility (VCI). Varicocele-related venous reflux and stasis, hyperthermia, hypoxia, and metabolic stress impair the electron transport chain, decrease mitochondrial membrane potential (ΔΨm) and adenosine triphosphate production, and increase mitochondrial reactive oxygen species (mtROS). These changes favor mitochondrial DNA (mtDNA) oxidation and abnormal release. Endoplasmic reticulum stress and autophagy or mitophagy can modify the severity of mitochondrial injury. Effective mitophagy limits mtROS accumulation and mtDNA escape, whereas incomplete or stalled flux allows these signals to persist. in injured nucleated testicular cells, cytosolic oxidized mtDNA can engage the cGAS-cGAMP-STING-TBK1IRF3/NF-κB pathway and promote type I interferon and proinflammatory signaling. NLRP3 inflammasome activity requires NF-κB-dependent priming and an additional activation signal, which can include NEK7 engagement, P2 × 7 signaling, mtROS, or oxidized mtDNA. Assembly of the NLRP3-ASC-caspase-1 complex promotes interleukin-1β and interleukin-18 maturation and gasdermin D-mediated pyroptosis. cGAS-STING and NLRP3 are presented as a potentially cross-amplifying network rather than a fixed linear cascade. Downstream consequences include programmed cell death, blood-testis barrier disruption, Sertoli and Leydig cell dysfunction, remodeling of the testicular immune microenvironment, sperm mitochondrial dysfunction, sperm DNA fragmentation, and impaired spermatogenesis. Changes in NAD+, acetyl-CoA, α-ketoglutarate, and S-adenosylmethionine may connect mitochondrial stress with DNA methylation, chromatin and protamine packaging, noncoding RNA regulation, and m6A modification. Persistence of these alterations after repair remains hypothetical. Varicocele repair remains the clinical foundation. Source-resolved biomarkers and pathway-directed adjunctive targets require longitudinal validation and reproductive-safety assessment. Solid arrows indicate relationships supported by experimental VCI or related mechanistic models; dashed arrows indicate proposed relationships that remain incompletely validated in human VCI

The main contribution of this review is therefore a disciplined working model, not a new clinical pathway. It specifies where evidence is direct, where it is borrowed from related models, and which experiments can close the gaps. Priority studies should combine source-resolved human sampling, longitudinal follow-up, cell-specific perturbation, and reproductive endpoints. Varicocele repair remains the clinical foundation. Biomarkers and pathway-directed treatments should enter practice only if they add reproducible prognostic or treatment value and improve pregnancy or live-birth outcomes without compromising reproductive safety (Fig. 1).

## Data Availability

No datasets were generated or analysed during the current study.

## Abbreviations

8-OHdG: 8-hydroxy-2′-deoxyguanosine
acetyl-CoA: Acetyl coenzyme A
ACSL4: Acyl-CoA synthetase long-chain family member 4
ASC: Apoptosis-associated speck-like protein containing a caspase recruitment domain
ATF4: Activating transcription factor 4
ATF6: Activating transcription factor 6
ATG7: Autophagy-related protein 7
ATP: Adenosine triphosphate
BAX: BCL2-associated X protein
BCL-2: B-cell lymphoma 2
BTB: Blood–testis barrier
CARD: Caspase activation and recruitment domain
cGAMP: Cyclic guanosine monophosphate–adenosine monophosphate
cGAS: Cyclic GMP-AMP synthase
CHOP: CCAAT/enhancer-binding protein homologous protein
Comet: Single-cell gel electrophoresis assay
CpG: Cytosine-phosphate-guanine dinucleotide
DAMPs: Damage-associated molecular patterns
DFI: DNA fragmentation index
CARD: Caspase activation and recruitment domain
DNMT3A: DNA methyltransferase 3A
DNMT3B: DNA methyltransferase 3B
ER: Endoplasmic reticulum
ETC: Electron transport chain
GPx: Glutathione peroxidase
GPX4: Glutathione peroxidase 4
GSDMD: Gasdermin D
GSDME: Gasdermin E
GSH: Glutathione
IL-1β: Interleukin-1β
IL-18: Interleukin-18
IRE1: Inositol-requiring enzyme 1
IRF3: Interferon regulatory factor 3
JNK: c-Jun N-terminal kinase
LC3: Microtubule-associated protein 1 light chain 3
LC3-I: Cytosolic form of microtubule-associated protein 1 light chain 3
LC3-II: Lipidated form of microtubule-associated protein 1 light chain 3
MDA: Malondialdehyde
MIR210HG: MIR210 host gene
MLKL: Mixed lineage kinase domain-like protein
MLLT4-AS1: MLLT4 antisense RNA 1
mtDNA: Mitochondrial DNA
mtROS: Mitochondrial reactive oxygen species
NAD+: Nicotinamide adenine dinucleotide
NEK7: NIMA-related kinase 7
NF-κB: Nuclear factor kappa B
NLRP3: NOD-like receptor family pyrin domain-containing 3
P2×7: Purinergic P2X receptor 7
PERK: Protein kinase R-like endoplasmic reticulum kinase
p-IRF3: Phosphorylated interferon regulatory factor 3
p-TBK1: Phosphorylated TANK-binding kinase 1
PLOD2: Procollagen-lysine, 2-oxoglutarate 5-dioxygenase 2
PROK2: Prokineticin 2
pro-IL-1β: Pro-interleukin-1β
RIPK1: Receptor-interacting serine/threonine-protein kinase
ROS: Reactive oxygen species
SAM: S-adenosylmethionine
SCD: Sperm chromatin dispersion
SCSA: Sperm chromatin structure assay
SDF: Sperm DNA fragmentation
SLC7A11-AS1: SLC7A11 antisense RNA 1
SOD: Superoxide dismutase
STING: Stimulator of interferon genes
TAC: Total antioxidant capacity
TBK1: TANK-binding kinase 1
TUNEL: Terminal deoxynucleotidyl transferase-mediated dUTP nick-end labeling
UPR: Unfolded protein response
VC: Varicocele
VCI: Varicocele-induced infertility
XBP1s: Spliced X-box-binding protein 1
α-KG: α-ketoglutarate
ΔΨm: Mitochondrial membrane potential
